# Microalgae-Derived Pigments: A 10-Year Bibliometric Review and Industry and Market Trend Analysis

**DOI:** 10.3390/molecules25153406

**Published:** 2020-07-28

**Authors:** Samara C. Silva, Isabel C. F. R. Ferreira, Madalena M. Dias, M. Filomena Barreiro

**Affiliations:** 1 Centro de Investigação de Montanha (CIMO), Instituto Politécnico de Bragança, Campus Santa Apolónia, 5300-253 Bragança, Portugal; samaras@ipb.pt (S.C.S.); iferreira@ipb.pt (I.C.F.R.F.); 2 Laboratory of Separation and Reaction Engineering—Laboratory of Catalysis and Materials (LSRE-LCM), Faculdade de Engenharia, Universidade do Porto, Rua Dr. Roberto Frias s/n, 4200-465 Porto, Portugal; dias@fe.up.pt

**Keywords:** microalgal pigments, phycocyanin, chlorophylls, astaxanthin, β-carotene, biorefinery, sustainable productive chains, microalgae biotechnology, market trends

## Abstract

Microalgae productive chains are gaining importance as sustainable alternatives to obtain natural pigments. This work presents a review on the most promising pigments and microalgal sources by gathering trends from a 10-year bibliometric survey, a patents search, and an industrial and market analysis built from available market reports, projects and companies’ webpages. The performed analysis pointed out chlorophylls, phycocyanin, astaxanthin, and β-carotene as the most relevant pigments, and *Chlorella vulgaris*, *Spirulina platensis*, *Haematococcus pluvialis*, and *Dunaliella salina*, respectively, as the most studied sources. *Haematococcus* is referred in the highest number of patents, corroborating a high technological interest in this microalga. The biorefinery concept, investment in projects and companies related to microalgae cultivation and/or pigment extraction is increasingly growing, particularly, for phycocyanin from *Spirulina platensis*. These pieces of evidence are a step forward to consolidate the microalgal pigments market, which is expected to grow in the coming years, increasing the prospects of replacing synthetic pigments by natural counterparts.

## 1. Introduction

Microalgae are the primary components of several ecosystems found in marine and freshwater. These organisms are catalogued in 72,500 species and 16 classes. The largest groups are green algae (*Chlorophyceae*), golden algae (*Chrysophyceae*), and diatoms (*Bacillariophyceae*). Cyanobacteria (*Cyanophyceae*), green algae, and diatoms are the most commonly explored for biotechnological purposes [1].

Microalgae are excellent sources of high-added value compounds with health benefits, presenting great potential in diverse markets. These include proteins, pigments, polyunsaturated fatty acids, lipids, vitamins, minerals, and polysaccharides. The global interest to explore microalgae-based products has been increasing due to their high-value biological compounds and abundant availability in the aquatic systems [2,3]. Geographically, the leading producers of microalgae biomass are in Taiwan, the United States, Japan, China, Spain, Brazil, Israel, Myanmar, and Germany, comprising an annual production of dry biomass of 19,000 tons, and generating a revenue of about USD 5.7 billion [4].

Among the wide variety of compounds from microalgae, natural pigments are one of the most relevant groups to be explored. Besides their colouring potential, natural pigments from microalgae have health benefits (antioxidant, anticancer, and anti-inflammatory) and can replace artificial colourants with advantages [5]. Three classes of pigments are found in microalgae: phycobiliproteins (until 8% of dry weight), carotenoids (usually 0.1–0.2% of dry weight, but achieving up to 14% in some species), and chlorophylls (0.5–1.0% of dry weight) [3,6].

The main objective of this work was to examine the current status and future perspectives of the microalgal pigments field. The work involves a bibliometric review based on Scopus database (10 years, 2009–2019), a patents search, and an analysis of worldwide trends based on available market reports, projects, and companies’ webpages. This allowed producing a systematic bibliographic review grounded on articulated evidences that pointed out the most studied pigments, their most promising sources, the technological maturity of some processes, industrial interest and implementation, and market proximity. In this context, bibliometric analysis provides an effective methodology of statistically evaluating published literature (e.g., articles, books or book sections), providing a way to measure their impact and identify trends in specific fields. This approach can also be used to access the influence of institutions, research groups, individual researchers, and countries [7,8]. Bibliometric analysis directed to evaluate specific scientific fields is also emerging. Examples include topics like microalgae [9,10], food [11,12], medicine [8,13], and environment [14]. To date, no study has been conducted on the potential of microalgae as a source of pigments for colourant applications, which is a relevant and emerging topic within microalgae biorefinery and of high industrial interest.

## 2. Results

### 2.1. Bibliometric Analysis

#### 2.1.1. Descriptive Analysis of the Bibliometric Data

The descriptive analysis of the gathered data from 2009 to 2019, using “microalgae” and “pigments” in Scopus database, is summarized in Table 1. In this search, 1177 documents and 4240 authors were identified. Briefly, 4211 were authors of multiauthored papers, and 29 were authors of single-authored documents. The author/documents ratio was 3.6 and the coauthor/documents ratio 4.98, whereas the documents/author ratio was 0.28.

**Figure 1:**
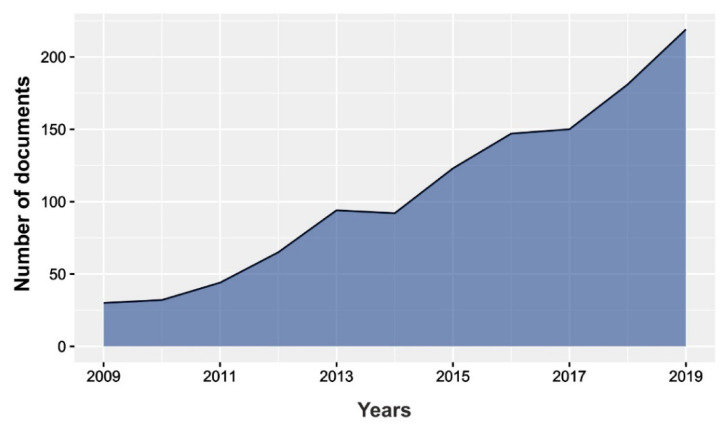
Evolution of the number of publications from 2009 to 2019 in the field of pigments from microalgae.

Figure 1 shows the evolution of the number of publications per year along the last 10 years period. It is possible to observe an increase in the number of publications in the periods 2009 to 2013, 2014 to 2016, and 2017 to 2019, while in 2013–2014 and 2016–2017 periods, the number of publications was maintained. The results showed an 80% increase in the number of publications dealing with microalgae and pigments from 2009 to 2019, which corroborates the increased interest in the field of microalgal pigments.

**Figure 2:**
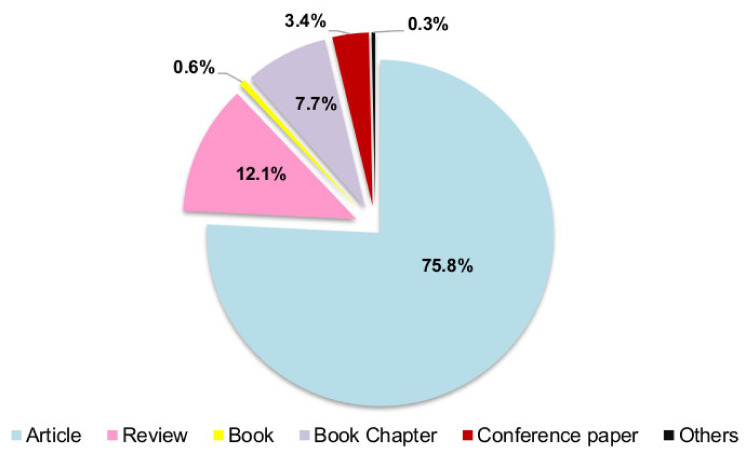
Distribution of different types of documents.

The different types of documents are presented in Figure 2 Most published documents are research articles (75.8%) and reviews (12.1%). Other documents appearing in less frequency are book chapters (7.7%), books (0.6%), conference papers (3.4%), and others (conference reviews, editorials, notes, and retracted documents; 0.3%). Based on these results, it is possible to conclude that the research published in this field is dispersed among several types of publications, showing a wide spreading of the thematic.

**Figure 3:**
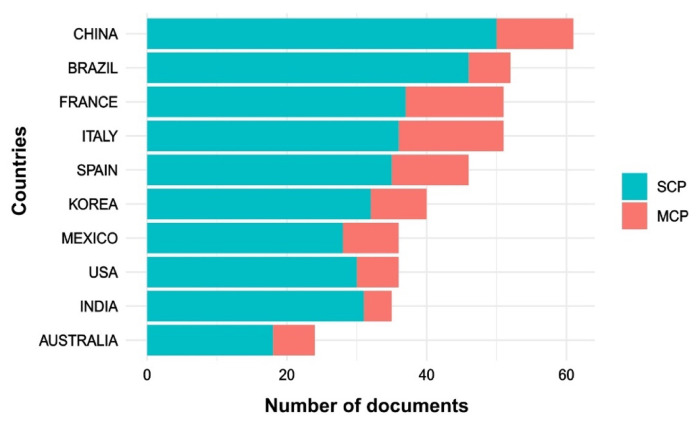
Most productive countries in the field of pigments from microalgae (SCP: single country publications, MCP: multiple country publications).

The name of countries was extracted from the analysed publications and the most productive ones were obtained from the analysis with the bibliometrix R-package. This analysis is essential to highlight which countries are the ones with high implementation in the microalgal-pigments research field. China, Spain, Brazil, the United States, Japan, Taiwan, Israel, and Germany are the leading producers of microalgae biomass and derived products [4]. Some of these countries are among the top 10 most productive countries in the microalgal pigments field (Figure 3).

China, Brazil, and France head the list of single-country publications, and Italy, France, and Spain are at the top in the multiple-country publications. These results corroborate the main indications stressed out from the bibliometric review of Garrido-Cardenas et al. [9] (research field “microalgae”), which pointed out the United States, China, Spain, France, Australia, and India among the 10-most productive countries in the microalgae field.

#### 2.1.2. Keyword Co-Occurrence Network Analysis

**Figure 4:**
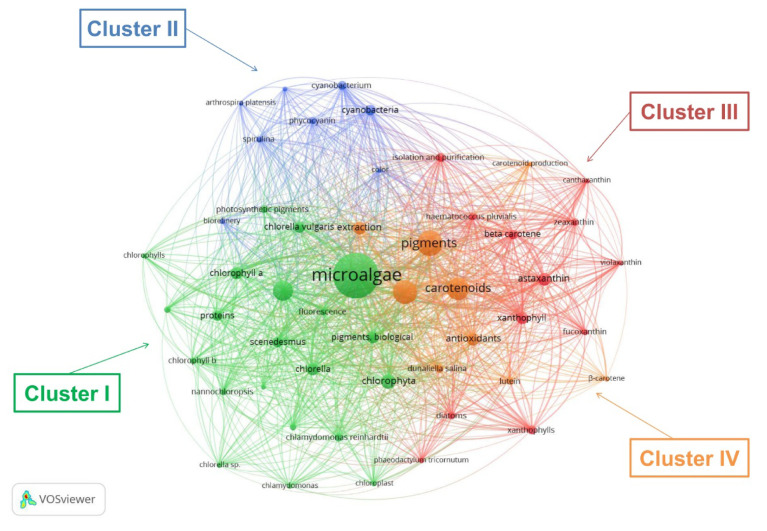
Keyword co-occurrence network analysis and emerging clusters (**Cluster I**: 21 words “**Chlorophylls** from *Chlorella vulgaris*”; **Cluster II**: 8 words “Phycocyanin from *Spirulina platensis*”; **Cluster III**: 12 words “Astaxanthin from *Haematococcus pluvialis*”; **Cluster IV**: 9 words “β-carotene from *Dunaliella salina*”).

To provide an overview of the research fields related to the words “microalgae” and “pigments”, the keyword co-occurrence was analysed. The achieved results are presented in Figure 4 (network visualization). Each circle represents a keyword with a minimum occurrence of 20-fold, and the respective size reflects the number of the occurrences. The higher the number of occurrences, the larger the keyword circle. The occurrences attribute indicates the number of documents in which a keyword appears. Clusters identify a set of related items and are reflected in the map using different colours. Although 198 terms met the preset requirements, only 50 items were considered relevant and analysed in the keyword co-occurrence network.

From the obtained results, there were 50 items organized in 4 clusters, 960 links and 8151 for total link strength. The keywords (listed in Table 2) were grouped into 4 main clusters, which substantiate 4 main fields related to pigments from microalgae.

Cluster I refers words such as microalgae, chlorophyll, photosynthesis, and *Chlorella*. Besides, green algae (*Chlamydomonas reinhardtii* and *Scenedesmus* genus), microalgae (*Chlorella vulgaris*, *Chlorella sorokiniana*, and *Nannochloropsis* genus) are also within this cluster. Although several microalgae are listed, *Chlorella vulgaris* is the one with the highest number of occurrences. Thus, this cluster highlights chlorophyll production from *Chlorella vulgaris*. Cluster II refers *Spirulina platensis* (*Arthrospira platensis*) and its major pigment, phycocyanin. Cluster III presents a microalga (*Haematococcus pluvialis*) and a diatom (*Phaeodactylum tricornutum*), with the microalga receiving the high number of occurrences. Consequently, this cluster put evidence in xanthophylls, being astaxanthin the most relevant pigment in number of occurrences. The major related microalga source is *Haematococcus pluvialis*. Other carotenoids, including fucoxanthin, a pigment that currently arouses great interest, due to its relevant biological functions and abundance in nature [15], was identified in this cluster. Cluster IV encompasses carotenes, namely, β-carotene and lutein from *Dunaliella salina*, but with higher relevance for the first pigment. The word biorefinery, more markedly presented in cluster II, which evidences phycocyanin from *Spirulina*, the field approaching more this concept, is also perceived in the context of other microalgae sources, namely, *Dunaliella salina*, *Haematococcus pluvialis*, *Chlorella*, and *Nannochloropsis*. These facts put in evidence the emerging interest in using the biorefinery concept in the microalgae field, where the combination of several products in one valorisation chain is needed.

## 3. Most Promising Microalgae-Derived Pigments: An Overview

Microalgae are recognized as primitive organisms since they are one of the oldest living forms on earth, existing in oceans since the beginning of earth’s formation, about 3 billion years ago [16]. Microalgae are photosynthetic organisms and include both prokaryotic (e.g., cyanobacteria: *Spirulina* sp.) and eukaryotic cells (e.g., green algae: *Chlorella* sp.). Their size ranges in diameter from 0.2 to 2.0 µm. Although these microorganisms are photoautotrophic, they can also grow under photoheterotrophic, heterotrophic or mixotrophic conditions [3,17]. They are grown in two different systems: open cultivation (lakes and ponds) and photobioreactors. Open cultivation is the oldest used method (since 1950); however, photobioreactors have increasing their use due to the ability to avoid environmental conditions and contamination [18]. Microalgae have a simple morphology, and can grow in mild conditions of light, salinity, pH, temperature, and nutrient concentration, or in extreme conditions (high or low temperatures, high or low CO_2_, exposure to high ultraviolet radiation and heavy metal concentrations) [3,10].

Currently, microalgae biomass is used in a wide range of applications, which include human ingestion, animal feed, cosmetics, pharmaceuticals, foods, bioenergy production, CO_2_ mitigation, nitrogen-fixing, and wastewater treatment. Among several microalgae species, the ones having higher commercial importance are *Spirulina* sp., *Chlorella* sp., *Haematococcus* sp., *Dunaliella* sp., *Botryococcus* sp., *Phaeodactylum* sp., *Porphyridium* sp., *Chaetoceros* sp., *Crypthecodinium* sp.; *Isochrysis* sp., *Nannochloris* sp., *Nitzschia* sp., *Schizochytrium* sp., *Tetraselmis* sp., and *Skeletonema* sp. [16]. *Spirulina platensis*., *Chlorella vulgaris*, *Dunaliella salina*, and *Haematococcus pluvialis* are the species cultivated at larger scale for dried biomass and/or pigment production [19]. This statement is also confirmed by the performed keyword co-occurrence analysis (Section 2.1.2).

*Spirulina* sp. and *Chlorella* sp. are mainly used as feed and food supplements (powder, pills, and tablets) due to their high content in proteins, lipids, vitamins, and minerals. These microalgae are dominating the microalgae market, being used as a source of pigments, such as phycobiliproteins and carotenoids. *Dunaliella salina* and *Haematococcus pluvialis* are produced at industrial level to obtain carotenoids, especially β-carotene (vitamin A precursor) and astaxanthin (a potent antioxidant), respectively [20].

To obtain bioproducts from microalgae biomass (e.g., pigments, proteins, or carbohydrates) some sequential steps need to be followed, from the choice of the most suitable microalgae strain to cultivation methods and downstream processes (e.g., harvesting, pretreatment, extraction, and purification). At pilot scale, the cultivation method is considered successful if it is able to generate a large amount of microalgae-biomass rich in the target high-value compounds. Following, after the maturation stage, harvesting is conducted to separate biomass from the culture medium. The harvesting process comprises different technologies, including filtration, flotation, centrifugation, and sedimentation [21].

Pretreatment is the next step and is used to rupture the cell wall to increase biomolecules extraction efficiency. Besides cell wall composition, some basic criteria must be considered, such as the location of the desired pigments in the algal tissues and their stability, which differ among microalgal classes. To break the cell wall, mechanical and nonmechanical methods can be used. Mechanical methods comprise bead mill, pressing, high-pressure homogenization, microwave treatment, ultrasonication, autoclaving, and lyophilization. Nonmechanical methods include the use of acids, alkalis, osmotic shock, and enzymatic processes [2,22].

The extraction step depends on the target pigment and can be performed either by conventional or nonconventional methods. Conventional methods include the use of solvents (pure solvents or aqueous/organic mixtures) depending on the nature of the pigment. The solvent choice must be done regarding the inherent ability of the pigment to be dissolve and extracted, without interfering in its structure. For example, ethanol, acetone, methanol, n-hexane, diethyl-ether, and chloroform have been used to extract microalgal pigments in combination with different techniques (e.g., saponification, freezing/thawing, and heating). In addition to solvent extraction, supercritical fluid extraction (SFE), which uses CO_2_ as the extraction solvent, is also used as a green alternative. Nonconventional methods comprise the electrotechnological techniques, e.g., the pulsed electric field, which is catching high attention since it does not generate heat during processing. High-voltage electric discharges, moderate electric fields, and microwave/ultrasound-assisted extraction are other electric-assisted methods that can be used to extract target-pigments [22,23].

At large scale, some challenges need to be faced, namely, in what concerns the high associated costs and immaturity of the up/downstream processing. Currently, some approaches have been studied to surpass these constraints [24]. According to Jacob-Lopes et al. [4], process integration and biorefinery implementation are the steps to be followed to achieve economic viability, over process intensification, which focuses in lowering energy consumption, waste generation, and the ratio “equipment size”/“production capacity”. Costa et al. [25] also states that the biorefinery is the most suitable alternative to improve economic and environmental achievements due to the full valorisation of the microalgal biomass.

The biorefinery concept is an approach to produce various products (fuels, chemicals, materials, energy, food, and feed) from the conversion of biomass (organic residues, aquatic biomass, and energy crops). Microalgae are considered potential candidates for biorefinery because they are rich in a diversity of compounds. Although the microalgae biorefinery approach is new compared with petroleum-based technologies or even with the modern lignocellulosic biorefineries, the interest in this approach has increased. This fact is corroborated by the performed keyword co-occurrence analysis (Section 2.1.2), where the keyword biorefinery was clearly evidenced linked to *Spirulina* and phycocyanin in Cluster III [26,27]. Through biorefinery approach, processes generate minimal waste, thus causing lower environmental impact, contributing also to reduce the high costs associated to microalgae up/downstream processes. Therefore, microalgae biorefinery is a promising path to achieve a sustainable economy in microalgae-based productive chains [28].

### 3.1. Chlorophylls from Chlorella Vulgaris

Chlorophylls are photosynthetic green pigments present in algae, bacteria, and higher plants. It is a fat-soluble compound with bioactive properties such as antioxidant [29] and antimutagenic activities [30]. Moreover, this pigment can be extracted from microalgae biomass at an amount ranging from 0.5% to 1.5% (dry weight basis). Chlorophylls are tetrapyrroles with centrally bonded magnesium, which due to its colour and biological properties are attracting great commercial interest. This natural green pigment finds applications in food, cosmetic, and pharmaceutical sectors [31,32].

Although chlorophylls are recognized as natural pigments, there are disadvantages related to their use. This pigment is chemically unstable to pH conditions and is sensitive to heat and light [3]. Therefore, chlorophylls are commonly transformed into sodium copper chlorophyll, in which, magnesium is replaced by sodium or copper to obtain a compound more stable than the former chlorophyll. This chlorophyll derivative is called chlorophyllin and is widely used in the food industry as food additive and colourant [33].

There are several types of chlorophyll structures, such as chlorophyll a (blue-green colour), b (brilliant green), c (yellow-green), d (brilliant/forest green), and f (emerald green). Photosynthetic organisms predominantly present chlorophyll a and b, whereas chlorophyll c, d, and f are found exclusively in some microalgae species, algae, and photosynthetic bacteria. [3,34].

*Chlorella* is a group of unicellular green eukaryotic microalgae that grows in freshwater and can be found as a food supplement or additive (e.g., tablets, powder, and capsules) and feed (e.g., aquaculture), due to its high nutritional content. *Chlorella vulgaris* is one of the well-known species belonging to the *Chlorella* genus and was first described in 1890. These microalgae are able to convert sunlight energy into chemical energy during photosynthesis and require nutrients (e.g., carbon and nitrogen) for their growth. Besides, *Chlorella* has a rapid growth and resistance to harsh conditions [35,36].

*Chlorella* cells growing under normal conditions are rich in protein (40–60%) and lipids, including essential fatty acids, minerals, and vitamins [37]. Furthermore, these microalgae can contain two main types of chlorophylls (a and b) for which they can accumulate considerable amounts (up to 4.5% of dry weight), being their most abundant pigments. Due to its chlorophyll content, *Chlorella* is known as the “emerald food”. However, even at lower amounts, it also contains other pigments, such as astaxanthin, c-astaxanthin, beta-carotene, and lutein [2,3,35].

### 3.2. Phycocyanin from Spirulina Platensis

Phycobiliproteins are light-harvesting protein-pigments. They are located in the supramolecular phycobilisomes on the external surface of the thylakoid membrane, corresponding to 40–50% of the total soluble proteins. They are water-soluble molecules classified into three main classes: phycocyanin (PC; blue pigment), allophycocyanin (AP; light-blue pigment), and phycoerythrin (PE; red pigment). The differences among them are the chemical structure, colour, and absorption spectra. Phycobiliproteins are composed of chromophore-bearing polypeptides with α and β subunits of around 20 kDa. They are used as fluorescent markers and show functional properties [38,39,40].

*Spirulina* (*Arthrospira*) sp. (phycocyanin) and *Porphyridium* sp. (phycoerythrin), cyanobacteria species, are the two most well-known microalgal sources for commercial production of phycobiliproteins [3,41,42]. *Spirulina* biomass has a high nutritional value and high potential for value-added biocompounds extraction. It is recognized as a source of vitamins (B1, B2, B12, E, and provitamin A), minerals (Fe, Mg, Ca, P, Cr, Cu, Na, and Zn), pigments (phycocyanin, chlorophylls, and carotenoids), essential fatty acids (γ-linolenic acid), phenolic compounds, biopeptides, and enzymes [43]. These compounds show several functional effects, acting as immunomodulators [44], antioxidant, and anti-inflammatory agents [45,46,47], anticancer and antimicrobials [48], and also show prebiotic effects [49].

Among the value-added products from *Spirulina*, there is the natural blue pigment called phycocyanin, the major phycobiliprotein found in this microalga [50]. This pigment was approved to be added into food matrices as a colour additive by the Food and Drug Administration in 2013 [51], being currently used as a natural colorant in foods such as beverages and confectionery products. Moreover, it finds applications in the pharmaceutical area due to therapeutic effects (antioxidant, anti-inflammatory, and anticancer activities), in the cosmetic industry (e.g., lipstick and eyeliners), and as fluorescent markers [39,52,53].

Phycocyanin is already commercially produced from *Spirulina platensis*, which is the most relevant source (up to 25% of the dry biomass, *w*/*w*) [53]. For phycocyanin production, photoautotrophic cultures of *S. platensis* grown in open raceways are used. This phototrophic production is commonly influenced by light (intensity, source, and wavelength). In addition, the culture medium should be appropriate with a significant amount of salt (e.g., carbonate and bicarbonate) and alkalinity (pH 9.5–9.8) [25,54]. According to the study of Chen et al. [55], an increase in the light intensity, from 100 to 700 µmol/m^2^/s, lead to a significant increase on both biomass and phycocyanin productivity. Photobioreactors have been used for *Spirulina* cultivation, being preferred over open raceways due to the lower risk of contamination and higher biomass productivity (5–20-fold higher productivity compared to the obtained in the opens raceways) [56].

### 3.3. β-Carotene from Dunaliella salina

Carotenoids are a group of compounds possessing colours from red and brown to orange and yellow. They share a common structure of isoprenic units and are divided into carotenes and xanthophylls. The differences between them are the functional groups and the base chemical structure. Carotenes are pure hydrocarbon compounds (e.g., β-carotene (C_40_H_56_)), while xanthophylls have oxygenated derivates in their structure, being hydroxyl and ketone groups at the end rings (e.g., astaxanthin (C_40_H_52_O_4_)). Due to the presence of oxygenate derivates (–OH and –CO), xanthophyll is a relatively hydrophilic compound [57,58,59]. Carotenoids can be applied in different fields. Examples include food colouring additives (e.g., β-carotene (EC160)) and nutraceuticals due to their role as active ingredients, cosmetics, pharmaceuticals, and animal feed (e.g., aquaculture of salmon). Besides, carotenoids are recognized as potent antioxidants and have health-promoting functional properties, such as decreasing triglycerides and increasing HDL (high-density lipoprotein) cholesterol [60], as well as preventing cancer [61].

Beta-carotene is the most prominent carotenoid. It is stored in the lipid globules located in the interthylakoid spaces of the chloroplast [62]. This pigment is recognized as a primary carotenoid. It is directly involved in photosynthesis due to their structure and functional properties of the photosynthetic apparatus. Furthermore, they are light-harvesting pigments and also photoprotective. The natural form of β-carotene comprises a mixture of two isomers (all-*trans* and 9-*cis*-*trans*), which are hard to obtain synthetically. Natural β-carotene has shown anticancer and antioxidant properties, helping to protect human and animal skin against photoaging and it effectively helps to control cholesterol levels decreasing the risk of cardiovascular diseases. In addition, the natural form of β-carotene is better absorbed by the human body, comparatively with the synthetic β-carotene form [3,58,63]

This lipid-soluble pigment is also known as a vitamin A precursor, which is biosynthesized by the human body. Beta-carotene is transformed enzymatically into retinal and then into retinol (vitamin A). It has a high provitamin A activity due to the production of two molecules of retinol from one of β-carotene [3,64]. Currently, β-carotene is extensively used as a natural colouring agent in the food industry (e.g., soft drinks, baked foods, and margarine), as well as an active ingredient in antioxidant supplements [65].

*Dunaliella salina* is a halotolerant green microalga and the richest microalgae source of β-carotene. *D. salina* may accumulate a high amount of β-carotene (up to 10%, dry biomass basis) under stressful conditions, such as high salinity/light intensity, extreme temperatures, and/or lack of nutrients [62,66,67]. For β-carotene production, *D. salina* cultivation is performed using a two-stage strategy. The first stage is known as the “greening stage”, where adequate conditions for *D. salina* growth are provided. After cells concentration reach a certain level, stress conditions are applied to accumulate more carotenoids, turning the colour of microalgae from green to orange (reddening phase) [57]. Beta-carotene from *Dunaliella salina* is approved as food colourant (E160 a (iv) Algal Carotenes) by European Commission [68].

### 3.4. Astaxanthin from Haematococcus pluvialis

Astaxanthin (3,3′-dihydroxy-β-carotene-4,4′-dione) is a fat-soluble orange-red pigment belonging to the carotenoids group as β-carotene (see Section 3.3). It is synthesized by some plants, algae, and bacteria. It can be also found in some fishes, crustaceans, and birds by accumulation due to food chains in nature [69,70]. Astaxanthin is considered the most potent antioxidant in nature, with an antioxidant activity 10-fold stronger than the ones of other carotenoids such as lutein, zeaxanthin, β-carotene, and canthaxanthin. Moreover, studies reported that this pigment enables protection against ultraviolet radiation and prevents oxidation of essential polyunsaturated fatty acids, besides showing neuroprotective activities [71,72,73].

Beta-carotene is a primary carotenoid, whereas astaxanthin is a secondary carotenoid. It is synthesized in the chloroplast and accumulated in the cytoplasm. The overproduction of secondary carotenoids is induced by environmental factors (e.g., oxidative stress, high salt concentration, light intensity, nutrient starvation, and temperature changes). They act as photoprotective pigments in response to the needed adaptation [3]. Aquaculture was one of the first applications for this pigment where it was applied as a feed additive to give the red colour to the flesh and shell of salmon, trout, shrimps, and langoustines. Astaxanthin is also used in dietary supplements due to health benefits such as anticancer, anti-inflammatory and anti-ageing effects [74,75]. Astaxanthin is available from chemical sources (synthetically produced) and from natural sources (extracted from microalgae, yeast, and crustaceans). Synthetic astaxanthin has a 20-fold lower antioxidant capacity than the natural form and corresponds to 95% of the astaxanthin available in the market. Only natural astaxanthin is approved for human consumption by the FDA [57,58,76].

*Haematococcus pluvialis* is a unicellular green freshwater microalga that can accumulate high amounts of astaxanthin (up to 3.8–5% of the microalga dry weight). This microalga is commonly used as a component of nutraceuticals, pharmaceuticals, aquaculture, food products, and cosmetics [37]. Like β-carotene production from *D. salina*, the same requirements are needed for astaxanthin from *H. pluvialis*. These two microalgae need to be in extreme conditions to produce and accumulate pigments [77]. Under stress conditions (e.g., nitrogen and phosphorus starvation, high solar intensities/temperatures, and salt stress), astaxanthin is accumulated in the lipid globules of the microalga cytoplasm [58,78]. To produce astaxanthin, there are four types of cell morphologies, namely, macrozooid (zoospores), microzooid, palmella, and haematocyst (aplanospores). In the first stage (green motile), the cells have macrozooid, microzooid, and palmella forms. However, in the second stage (red nonmotile), the cells are aplanospores. Therefore, cells change their colour from greenish to reddish [79].

The European Commission authorized the use of astaxanthin-rich oleoresin from *Haematococcus pluvialis* in food supplements at a maximum level of 8 mg (astaxanthin)/day [80]. In a recent scientific opinion published by the European Food Safety Authority (EFSA), the safety of astaxanthin from *Haematococcus pluvialis* as novel food ingredient was corroborated, and the intake of 8 mg of astaxanthin/day was confirmed as safe for adults [81].

## 4. Patents Analysis

After identifying the relevant fields with the keyword co-occurrence analysis, the patent analysis was conducted considering four groups of microalgae (*Spirulina*, *Chlorella*, *Haematococcus*, and *Dunaliella*) and their respective main pigments (phycocyanin, chlorophylls, astaxanthin, and beta-carotene, respectively).

**Figure 5:**
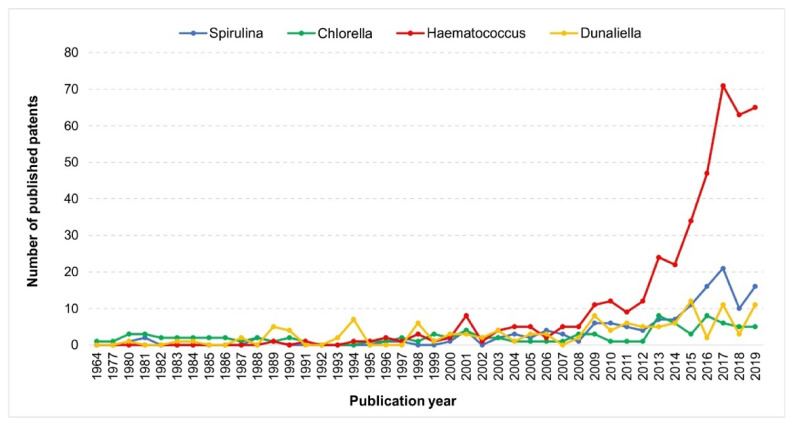
Evolution of the number of published patents over the years for each analysed microalga. Total number of published patents: *Haematococcus*: 418; *Spirulina*: 137; *Dunaliella*: 124, and *Chlorella*: 97. Source: Espacenet Patent database. Consulted on April 21, 2020.

The evolution of the number of published patents over the years, for each analysed microalga, is shown in Figure 5. *Chlorella* is the group of microalgae with the oldest issued patent, namely, the registration dates from 1964 [82]. Patents related to *Spirulina* and *Dunaliella*, and their respective pigments, emerged only 16 years later (1980), while for *Haematococcus*, only 25 years later (1989). Although *Chlorella* has the oldest published patent, there was not an increased trend in the number of published documents over the years. This fact can be correlated with the value chain of chlorophyll pigments, which can be obtained from a diversity of other natural sources (e.g., spinach).

*Dunaliella* related patents showed an increased tendency particularly in the recent years (2015, 2017, and 2019). Regarding *Spirulina* and *Haematococcus*, some interesting results were observed. From 2000 to 2019, an increase in the published number of patents was identified for both microalgae, but with a notorious preponderance of *Haematococcus* over *Spirulina*. In 2017, around 71 patents related to *Haematococcus*-astaxanthin were published, while only 21 addressed *Spirulina*-phycocyanin. This fact is also corroborated at an industrial level since *Haematococcus* as a source of xanthophylls has driven high interest.

Within the patent analysis, and in comparison with the other analysed microalgae, *Haematococcus*, and its pigment astaxanthin, was the uppermost cited (418 documents). Moreover, the first patent referring these microalgae dates from 1989 referring to the production of carotenoids (astaxanthin) [83]. The most recent one within the evaluated period was issued in December 2019 and details the development of a prawn feed with *Haematococcus* and astaxanthin (100–2100 mg) aiming at improving antioxidant and antistress activities. The observed increased industrial interest for *Haematococcus* and *Spirulina* as source of pigments can be justified by the particularities of these two pigments (astaxanthin and phycocyanin); phycocyanin is the only natural blue sources, and astaxanthin is the most potent antioxidant in nature.

*Spirulina* microalga was the one with the second most significant number of published patents (137 documents). The first published patent, entitled “Preparation of alcohol-resistant high-purity phycocyanin” was published in 1980, and describes the treatment of phycocyanin obtained from *Spirulina* by protease action to obtain a high-purity alcohol-resistant phycocyanin [84]. The most recent patent was published in December 2019 (i.e., Sterilization method of *Spirulina* powder with high phycocyanin content) and is directed to the development a sterilization method for the *Spirulina* powder to effectively preserve phycocyanin [85].

In Table 3, the details of some selected patents related to the four surveyed microalgae-derived pigments are presented. Most of the patents are related with the cultivation and respective pigment-extraction methods. For example, Cao et al. [86] from Jiangsu University of Science and Technology granted a patent concerning the increase of *Chlorella* chlorophyll content using a simple continuous operating mode. In addition, a patent focusing a method of coproducing phycocyanin, polysaccharides, and a protein-rich feed from fresh *Spirulina* was recently granted to two inventors Xiao and Zhang [87]. Patents directed to the use of microalgal pigments can be also found. This is the case of Yuanchuang Environmental Protection Technology Company, a patent describing a method for fabric dying using beta-carotene from *Dunaliella salina* was granted [88]. Moreover, the development of tablet candies using *Haematococcus pluvialis* astaxanthin was registered by Liu and Wei [89].

## 5. Industry and Market Trend Analysis

In order to evaluate recent trends on microalgae and pigments production, an overview analysis was performed based on available market reports, projects, and companies’ webpages. Namely, a high-value niche market related to microalgae biomass, as well as industries producing them at large scale, already exist. Examples include Cyanotech (www.cyanotech.com), Earthrise (www.earthrise.com), and Cellana (www.cellana.com) in the United States; AlgoSource (www.algosource.com) in France; Archimede Ricerche (www.archimedericerche.com) in Italy; BlueBioTech (www.bluebiotech.de) in Germany; Algaetech (www.algaetech.com.my) in Malaysia; Bluetec (www.bestphycocyanin.com) in China; AlgaEnergy (www.algaenergy.com) and Monzón Biotech (www.mznbiotech.com) in Spain; Spigreen (www.spigreen.com.br) in Brazil; and Necton (www.necton.pt) in Portugal. Moreover, a biotechnology company called A4F (www.a4f.pt), operating in Portugal, is specialized in the design, building, operation, and transfer of commercial-scale microalgae production, using different technologies. In addition, Algalife (www.alga-life.com) presents a novel perspective in the development of innovative pigments and fibres from microalgae (biotech textile).

Many projects related to the microalgae field have been approved and funded by the European Commission in the last years. Examples include the ongoing project entitled VALUEMAG (Horizon 2020) (www.valuemag.eu), which is related to the development of an advanced magnetic method for microalgae cultivation and MAGNIFICENT (Horizon 2020) (www.magnificent-algae.eu), an ongoing project aiming at transforming microalgae biomass into valuable ingredients for food, aquafeed, and cosmetic applications.

This growing interest in the exploitation of microalgae field is confirmed by a report published in 2019 by QYResearch’s. According to this report, the global algae market was USD 590 million by 2018. It is expected to reach USD 970 million by the end of 2025, growing at a CAGR (compound annual growth rate) of 6.7% between 2019 and 2025 [106]. Furthermore, it is also estimated that by 2025, the microalgae product’s market will become a large-scale business [107]. For the main microalgae-derived pigments, a worldwide commercial production of around 10,000 tons/year for *Spirulina*, 4000 tons/year for *Chlorella*, 1000 tons/year for *Dunaliella*, and 200 tons/year for *Haematococcus* is estimated [108]. Face to these results, it is possible to foresee, in the next years, an increase use of algae pigments in different industrial fields.

Following the recent trends, *Spirulina* is one of the most worldwide cultivated microalgae. Namely, according to a report published by Allied Market Research, the *Spirulina* market achieved USD 348 million in 2018. It is expected to reach USD 779 million by 2026, growing at a CAGR of 10.5% between 2019 and 2026. Concerning to geographical regions, North America was the leader of the global *Spirulina* market in 2018. It is estimated to remain dominant during 2019–2026 period. In this region, these results may be justified by the increasing demand for natural food colouring agents. The Asia-Pacific region is expected to grow at a CAGR of 11.2% over the forecasted period (2019–2026), providing profitable opportunities for market players [109].

Regarding *Spirulina*-pigment, phycocyanin, according to a report published by the Future Market Insights, the global market was valued at USD 112.3 million in 2018. By the end of 2025, it is estimated to reach a value surpassing USD 232.9 million, growing at CAGR of 7.6% during this period [110]. The growing interest in phycocyanin pigment is visible through the recently approved European project SPIRALG (Horizon 2020) (www.spiralg.eu) related to the development of a demonstration plant for phycocyanin production, where the valorisation of the generated co-products supports sustainability. Moreover, Naturex (www.naturex.com), a company specialized in natural speciality ingredients for food, health, and cosmetics, now a Givaudan business (the major flavour and fragrances producer), announced to triple the phycocyanin production through a new extraction line. This blue-pigment from *Spirulina* is already used in the food/cosmetic industry, namely, in beverages (e.g., Bloo Tonic^®^, B-blue *Spirulina* drink, and M&Ms^®^ chocolates).

Phycocyanin can also be used, either as a food supplement or colourant due to its functional benefits. In this context, AlgoSource (www.algosource.com), a French company, produces phycocyanin as a liquid extract (Spirulysat^®^) that is sold in phials (10 mL). Another company called Spira (www.spirain.com) produces phycocyanin in powder form (Electric Sky^®^) to be used for functional health benefits (food supplement) and colouring applications. These trends are also consubstantiated in the scientific literature that reports, in some studies, the addition of phycocyanin to food as a bioactive natural colourant. For example, Mohammadi-Gouraji, Soleimanian-Zad, and Ghiaci [111] developed a phycocyanin-enriched yogurt and showed positive effects in colour stability. A gelatine-based film incorporated with phycocyanin from *Spirulina* sp. was developed by Chentir et al. [112], proving to be a promising coloured external or inner packaging.

From the keyword co-occurrence results, *Chlorella* is also a group of microalgae raising an increased interest in recent years. According to a report published by Market Research Future, the global *Chlorella* market is estimated to reach USD 210.15 million by 2024 at a 6.35% CAGR between 2019 and 2024. Furthermore, in 2018 Europe, had the major market sharing (40%) of the global *Chlorella* market. This trend is expected to remain throughout the period 2019–2014 [113]. This report also refers two European countries as holding *Chlorella* major market sharing in 2019, Germany and the United Kingdom. This is already evidenced by the company Sun *Chlorella* (www.sunChlorella.com) in the United Kingdom that produces *Chlorella* in tablets, granules, and powder forms, as well as “W Sun Gold” a *Chlorella* extract blended with malic acid and “Sun *Chlorella* Cream” a skincare cream. Additionally, researchers from Portugal have developed a study dealing with the adding of *Chlorella vulgaris* biomass to traditional butter cookies as a colouring agent, with positive results concerning colour stability evaluated along a storage period of three months [114].

For chlorophylls, according to the report published by Value Market Research, the global market of chlorophyll extract was valued at USD 279.5 million in 2018 and is estimated to reach USD 463.7 million by 2025, with a CAGR of 7.5% from 2018 to 2025 [115].

The last two microalgae found in the keyword co-occurrence results were *Haematococcus pluvialis* and *Dunaliella salina*. The production of *H. pluvialis* is about 300 tons per year in the United States, Israel, and India, and several commercial companies are involved in the large-scale production of this microalga. Examples include Cyanotech Corporation (www.cyanotech.com), Algatech (www.algatech.com), and AstaReal (www.astareal.se) [37,76]. The first company with a thriving commercial production of astaxanthin was AstaReal Group (www.astareal.com) in 1994. Its product, AstaReal^®^ Astaxanthin, is sold in three different forms, AstaReal^®^ oleoresin, AstaReal^®^ EL25, and AstaReal^®^ A1010. In Brazil, a company called Ocean Drop (www.oceandrop.com.br) sells cosmetic products incorporated with astaxanthin (e.g., face serum, body and face hydration creams Antiox^®^), and researchers from Japan [116] have developed bread with astaxanthin from *Haematococcus pluvialis* founding that astaxanthin was well preserved from degradation during baking.

The commercial production of *D. salina* started in the 1980s in Australia, Israel, and the United States. Western Biotechnology and Betatene (Australia) were the first producers of this microalga for β-carotene production. Nature Beta Technologies (NBT) started the production at the same time in Israel. Western Biotechnology and Betatene were acquired by Cognis Nutrition and Health and are currently owned by BASF (www.nutrition.basf.com), the major producer of *Dunaliella* natural β-carotene in the world. Still today, *D. salina* has been produced and its global production per year is estimated to be 1200 tons (dry weight) [37,117].

In 2018, the global astaxanthin market exceeded USD 600 million and is estimated to reach USD 800 million by the end of 2026 at a growth over 3.5% CAGR from 2019 to 2026, according to the report published by Global Market Insights [118]. Regarding β-carotene, the global market size is expected to achieve USD 618.94 million by the end of 2026 and grow at a CAGR of 3.8% from 2018 to 2026, according to MarketWacth report of 2019 [119]. Moreover, the interest in this pigment trough the funded European project d-Factory must be highlighted (The microalgae biorefinery, 2013–2017) (www.d-factoryalgae.eu). The main objective of this project was to produce nutraceuticals, chemicals, feed, and fuels from *D. salina*, using the biorefinery concept. ABACUS (Horizon 2020) is another European project related to the development of a new algal biorefinery with focus on carotenoids production (β-carotene, astaxanthin, and fucoxanthin) for nutraceuticals and cosmetics actives.

Summarizing, among the focused pigments (phycocyanin, chlorophylls, β-carotene, and astaxanthin), β-carotene and astaxanthin are the pigments with the most significant global market evolution by 2026, showing the great interest on carotenoids from natural sources. According to Mulders et al. [120], β-carotene and astaxanthin presented an increase in global pigments sales volume, indicating a change from chemical synthesis to natural production.

In fact, the increased interest in natural colourants can be associated to the toxic effects of synthetic counterparts and also to sustainability issues. Microalgae add value compared to other natural pigment sources (e.g., plants, fruits, and animal sources) because they do not require arable land, are cost-effective, and present higher productivities (fast grown rates). Moreover, microalgae can be also grown using wastewater/seawater and their biomass can be converted into several compounds as well as pigments (biorefinery concept) [22]. They are renewable, sustainable, and eco-friendly sources of pigments, bringing advantages to the industry (availability of natural pigments with a diverse pallet of colours) and to consumers (health benefits). Thus, natural microalgal pigments have a high potential to substitute synthetic counterparts.

## 6. Materials and Methods

### 6.1. Bibliometric Analysis

The scientific literature was searched using two words, “microalgae” and “pigments”, in the article title, abstract, and keywords. The objective was to avoid microalgae wide-scope articles and privilege pigments-centred works. The Scopus database (Elsevier) was used, and 1177 documents were found after limiting the search timescale from 2009 to 2019. The survey was conducted on February 13, 2020. The collected data was analysed for bibliometrics using the open-source RStudio software (www.rstudio.com) (v1.2.1335, April 4, 2019), with bibliometrix R-package [121].

The keyword co-occurrence network was constructed and visualized with the VOSviewer software, a freely available program (www.vosviewer.com) developed by van Eck and Waltman [122]. The keyword co-occurrence was screened to find the most commonly used keywords related to the searched words (microalgae and pigments). The analysis presents three options, namely, “author keywords” and “index keywords”, and “all keywords” being selected “all keywords”; then “full counting” was used as the counting method. The minimum number of occurrences of a keyword was 20, and manual inspection was used to exclude irrelevant keywords.

### 6.2. Patent Analysis

Based on the keyword co-occurrence results, a search in a patent database was conducted to find technological trends in the field of pigments from microalgae. Patent numbers were collected from Espacenet Patent Search (worldwide.espacenet.com) using the advanced search option (title or abstract) on 21 April 2020. From the results of keyword co-occurrence, 4 different thematics were found to be relevant to include in the patent search, namely, “*Spirulina* and phycocyanin”; “*Chlorella* and (chlorophylls or chlorophyll)”; “*Dunaliella* and (carotenoids or carotenoid or beta-carotene)”; “*Haematococcus* and (xanthophylls or xanthophyll or astaxanthin)”. The patent search was limited to patents published until 2019.

### 6.3. Industry and Market Trend Analysis

The industry and market trend analysis was done based on information freely available on the internet, namely, “Market reports”, “Project webpages”, and “Companies webpages”. The search was directed to the pigments and microalgae previously identified on the keyword co-occurrence results.

## 7. Conclusions

Phycocyanin, chlorophylls, β-carotene, and astaxanthin are the pigments receiving the highest research investment in the period 2009–2019. The species *Chlorella vulgaris*, *Spirulina platensis*, *Haematococcus pluvialis*, and *Dunaliella salina* emerged as the main microalgal sources for chlorophylls, phycocyanin, astaxanthin, and β-carotene, respectively. According to the bibliometric analysis, the most productive countries were China, Brazil, and France. *Haematococcus* concentrates the highest number of patents pointing out a higher maturity for this productive technology. Investment in projects, namely, European projects, and companies related to microalgae cultivation and/or pigment extraction, are becoming a reality with preponderance for phycocyanin from *Spirulina platensis*, which is also the sector where the biorefinery concept is gaining more relevance.

Concluding, all the discussed microalgae and derived pigments have a promising future. Microalgae have been recognized as natural colourant/nutraceuticals sources by the scientific community, and their potential has been already proved in several industrial fields. Although some challenges need to be faced and overcome, the pigments from microalgae will soon surpass synthetics forms due to their renewable and sustainable nature and added health benefits.

## Figures and Tables

**Table 1:** Descriptive analysis of retrieved data.

Descriptive Analysis	
Documents	1177
Period	2009–2019
Annual percentage growth rate	22.0
Average citations per documents	18.0
Authors	4240
Author appearances	5863
Authors of single-authored documents	29
Authors of multiauthored documents	4211
Documents per author	0.28
Authors per document	3.60
Coauthors per documents	4.98
Collaboration Index	3.68

**Table 2:** Clusters and words obtained from keyword co-occurrence network analysis.

Cluster	Words
Cluster I	21 words: cell disruption, *Chlamydomonas*, *Chlamydomonas reinhardtii*, *Chlorella*, *Chlorella* sorokiniana, *Chlorella* sp., *Chlorella vulgaris*, chlorophyll, chlorophyll a, chlorophyll b, chlorophyll content, chlorophylls, chlorophyta, chloroplast, fluorescence, microalgae, *Nannochloropsis*, photosynthetic pigments, pigments and biological, proteins and *Scenedesmus*. Theme: Chlorophylls from *Chlorella vulgaris*.
Cluster II	8 words: *Arthrospira platensis*, biorefinery, colour, cyanobacteria, cyanobacterium, phycocyanin, *Spirulina* and *Spirulina platensis*. Theme: Phycocyanin from *Spirulina platensis*.
Cluster III	12 words: astaxanthin, beta carotene, canthaxanthin, diatoms, fucoxanthin, *Haematococcus pluvialis*, isolation and purification, *Phaeodactylum tricornutum*, violaxanthin, xanthophyll, xanthophylls, zeaxanthin. Theme: Astaxanthin from *Haematococcus pluvialis*.
Cluster IV	9 words: antioxidants, carotenoid, carotenoid production, carotenoids, *Dunaliella salina*, extraction, lutein, pigments, β-carotene. Theme: β-carotene from *Dunaliella salina*.

**Table 3:** Details of patents in the field of pigments from microalgae.

Microalgae	Title	Earliest Priority	Applicants	Publication Date	Inventors	Patent Number	Reference
*Chlorella*	Method for increasing content of *Chlorella* chlorophyll	26/11/2018	Jiangsu University of Science & Technology	19/02/2019	Cao, Bei; Deng, Xiangyuan; Gao, Kun; Hu, Xiaoli; Li, Da; Yan, Runjiao;	CN109355194A	[86]
	*Chlorella* with high content of chlorophyll and carotenoid	30/09/2014	Yakult Honsha Kk	30/09/2014	Ishikawa, Eiji; Kasaba, Keiko; Shibata, Shinya; Shimura, Kisaku Shirota, Satoshi;	JP2016067313A	[90]
	Method for producing *Chlorella* powder with increased chlorophyll content and antioxidant activity	14/11/2012	Daegu Catholic University Industry Academic Cooperation Foundation	28/04/2014	Hong, Joo Heon; Kang, Il Jun; Lee, Dae Hoon; No, Hong Kyoon; Park, Hye Mi;	KR101389471B1	[91]
	Green color-based beverage composition	27/12/2006	Nippon Sangaria Beverage Company	17/07/2008	Kitamura, Hirofumi; Yokoi, Katsumi;	JP2008161094A	[92]
	Chlorophyll-rich and salt-resistant *Chlorella*	17/12/1998	Kirin Brewery Nisshin Oil Mills Ltd.	22/06/2000	Nakanishi, Koichi;	CA2355289A1	[93]
*Spirulina*	Method for industrial co-production of phycocyanin, *Spirulina* polysaccharide and protein feed from fresh *Spirulina*	30/12/2018	Haitao Xiao; Dezhi Zhang	09/04/2019	Xiao, Haitao; Zhang, Dezhi;	CN109593128A	[87]
	A separation and purification method for high-purity phycocyanin	17/10/2018	Yunnan University	15/01/2019	Wang, Zhengkun; Zhang, Hao; Zhu, Wanlong;	CN109206504A	[94]
*Spirulina*	Preparation method of high-purity phycocyanin	30/04/2014	China Pharmaceutical University	20/08/2014	Li, Jing; Ou, Yu; Yuan, Zhijun	CN103992402A	[95]
	Extraction process for *Spirulina* phycocyanin and use of *Spirulina* phycocyanin	10/09/2015	Yang Zhou	06/01/2016	Zhou, Yang;	CN105218664A	[96]
	Blue liquid *Spirulina* beverage and preparing process thereof	28/12/1995	Guangdong Marine Organisms Food	31/07/1996	Changlan, Zhang; Zhanmin, Wang; Zhongliang, Liu;	CN1127611A	[97]
*Dunaliella*	Dyeing method of natural *Dunaliella salina* beta-carotene	11/09/2018	Quanzhou Yuanchuang Environmental Protection Tech Co Ltd.	12/02/2019	Tang, Yuzhao;	CN109322180A	[88]
	Method for promoting accumulation of carotenoids and beta-carotene in *Dunaliella* by using beta-ionone	28/06/2018	South China University of Technology	07/12/2018	Jiang, Jianguo; Liang, Minghua; Wan, Lin;	CN108949888A	[98]
	*Dunaliella* variant and method for producing pigment by using same	01/06/2016	Industry-University Cooperation Foundation Hanyang University	07/12/2017	Jin, Eon Seon; Kim, Min Jae;	WO2017209510A1	[99]
	Method for preparing purity beta-carotene nano powder from *Dunaliella salina*	24/06/2011	National Chung Hsing University	01/01/2013	Chang, Chieh-Ming; Shen, Yi-Chen; Chang, Chieh Ming; Shen, Yi Chen;	TW201300157A	[100]
	High purity beta-carotene	27/10/1992	Natural Carotene Corporation	10/05/1994	Haigh, W Geoffrey;	WO9410140A1	[101]
*Haematococcus*	Method for rapidly extracting astaxanthin from *Haematococcus pluvialis*	31/07/2019	Hangzhou Nyushu Biological Tech Co. Ltd.	22/11/2019	Hong, Yinqiu; Wang, Ping;	CN110483358A	[102]
*Haematococcus*	*Haematococcus pluvialis* tabletted candies and preparation method thereof	17/01/2019	Nanning Fresh Life Biotechnology Co Ltd.	26/04/2019	Liu, Hua; Wei, Chunliu;	CN109673801A	[89]
	Method for inducing efficient accumulation of astaxanthin in *Haematococcus pluvialis* by adding nanometer material	19/10/2018	Graduate School Shenzhen Tsinghua University	01/02/2019	Gao, Manyu; Qian, Wei; Wang, Lu; Zhu, Xiaoshan;	CN109294920A	[103]
	Preparation method of antioxidant astaxanthin soft capsules	27/08/2018	Sun Yat Sen University	07/12/2018	Duan, Minghui; Fang, Ting; Ge, Fahuan; Ma, Jinfang; Shi, Qinglong;	CN108936666A	[104]
	Method to enhance the astaxanthin biosynthesis in microalga *Haematococcus pluvialis*	25/08/2016	Astabio Co Ltd. University Korea Res & Bus Found	07/03/2018	Choi, Yoon E; Lee, Chang Su;	KR20180023232A	[105]

## References

[1] Guiry M.D. (2012). How many species of algae are there?. J. Phycol..

[2] Khanra S., Mondal M., Halder G., Tiwari O.N., Gayen K., Bhowmick T.K. (2018). Downstream processing of microalgae for pigments, protein and carbohydrate in industrial application: A review. Food Bioprod. Process..

[3] Christaki E., Bonos E., Florou-Paneri P., Kim S.-K. (2015). Innovative Microalgae Pigments as Functional Ingredients in Nutrition. Handbook of Marine Microalgae: Biotechnology Advances.

[4] Jacob-Lopes E., Maroneze M.M., Deprá M.C., Sartori R.B., Dias R.R., Zepka L.Q. (2019). Bioactive food compounds from microalgae: An innovative framework on industrial biorefineries. Curr. Opin. Food Sci..

[5] Rodrigues D.B., Menezes C.R., Mercadante A.Z., Jacob-Lopes E., Zepka L.Q. (2015). Bioactive pigments from microalgae *Phormidium autumnale*. Food Res. Int..

[6] D’Alessandro E.B., Antoniosi Filho N.R. (2016). Concepts and studies on lipid and pigments of microalgae: A review. Renew. Sustain. Energy Rev..

[7] Kamdem J.P., Duarte A.E., Lima K.R.R., Rocha J.B.T., Hassan W., Barros L.M., Roeder T., Tsopmo A. (2019). Research trends in food chemistry: A bibliometric review of its 40 years anniversary (1976–2016). Food Chem..

[8] Iftikhar P.M., Ali F., Faisaluddin M., Khayyat A., De Gouvia De Sa M., Rao T. (2019). A Bibliometric Analysis of the Top 30 Most-cited Articles in Gestational Diabetes Mellitus Literature (1946–2019). Cureus.

[9] Garrido-Cardenas J.A., Manzano-Agugliaro F., Acien-Fernandez F.G., Molina-Grima E. (2018). Microalgae research worldwide. Algal Res..

[10] Sydney E.B., Schafranski K., Barretti B.R.V., Sydney A.C.N., Zimmerman J.F.D.A., Cerri M.L., Mottin Demiate I. (2019). Biomolecules from extremophile microalgae: From genetics to bioprocessing of a new candidate for large-scale production. Process Biochem..

[11] Rodríguez-Rojas A., Arango Ospina A., Rodríguez-Vélez P., Arana-Florez R. (2019). ¿What is the new about food packaging material? A bibliometric review during 1996–2016. Trends Food Sci. Technol..

[12] Yeung A.W.K., Mocan A., Atanasov A.G. (2018). Let food be thy medicine and medicine be thy food: A bibliometric analysis of the most cited papers focusing on nutraceuticals and functional foods. Food Chem..

[13] Bilik O., Damar H.T., Ozdagoglu G., Ozdagoglu A., Damar M. (2020). Identifying trends, patterns, and collaborations in nursing career research: A bibliometric snapshot (1980–2017). Collegian.

[14] Olisah C., Okoh O.O., Okoh A.I. (2019). Global evolution of organochlorine pesticides research in biological and environmental matrices from 1992 to 2018: A bibliometric approach. Emerg. Contam..

[15] Kraan S., Domínguez H. (2013). Pigments and minor compounds in Algae. Functional Ingredients from Algae for Foods and Nutraceuticals.

[16] Sathasivam R., Radhakrishnan R., Hashem A., Abd_Allah E.F. (2019). Microalgae metabolites: A rich source for food and medicine. Saudi J. Biol. Sci..

[17] Acién F.G., Molina E., Reis A.G., Torzillo G., Zittelli G.C., Sepúlveda C., Masojídek J., Gonzalez-Fernandez C., Muñoz R. (2017). Photobioreactors for the production of microalgae. Microalgae-Based Biofuels and Bioproducts.

[18] Manivasagan J.V.P., Kim S.-K., KIM S.-K. (2015). Marine Microalgae Biotechnology: Present Trends and Future Advances. Handbook of Marine Microalgae.

[19] Hu J., Nagarajan D., Zhang Q., Chang J.S., Lee D.J. (2018). Heterotrophic cultivation of microalgae for pigment production: A review. Biotechnol. Adv..

[20] Da Silva Vaz B., Moreira J.B., de Morais M.G., Costa J.A.V. (2016). Microalgae as a new source of bioactive compounds in food supplements. Curr. Opin. Food Sci..

[21] Tang D.Y.Y., Khoo K.S., Chew K.W., Tao Y., Ho S.H., Show P.L. (2020). Potential utilization of bioproducts from microalgae for the quality enhancement of natural products. Bioresour. Technol..

[22] Nwoba E.G., Ogbonna C.N., Ishika T., Vadiveloo A., Alam M.A., Xu J.-L., Wang Z. (2020). Microalgal pigments: A Source of Natural Food Colors. Microalgae Biotechnology for Food, Health and High Value Products.

[23] Poojary M.M., Barba F.J., Aliakbarian B., Donsì F., Pataro G., Dias D.A., Juliano P. (2016). Innovative Alternative Technologies to Extract Carotenoids from Microalgae and Seaweeds. Mar. Drugs.

[24] De Souza M.P., Hoeltz M., Gressler P.D., Benitez L.B., Schneider R.C.S. (2019). Potential of Microalgal Bioproducts: General Perspectives and Main Challenges. Waste Biomass Valorization.

[25] Costa J.A.V., Freitas B.C.B., Rosa G.M., Moraes L., Morais M.G., Mitchell B.G. (2019). Operational and economic aspects of Spirulina-based biorefinery. Bioresour. Technol..

[26] Das D. (2016). Introduction. Algal Biorefinery: An Integrated Approach.

[27] Chew K.W., Yap J.Y., Show P.L., Suan N.H., Juan J.C., Ling T.C., Lee D.-J., Chang J.-S. (2017). Microalgae biorefinery: High value products perspectives. Bioresour. Technol..

[28] Banu J.R., Kavitha S., Gunasekaran M., Kumar G. (2020). Microalgae based biorefinery promoting circular bioeconomy-techno economic and life-cycle analysis. Bioresour. Technol..

[29] Lanfer-Marquez U.M., Barros R.M.C., Sinnecker P. (2005). Antioxidant activity of chlorophylls and their derivatives. Food Res. Int..

[30] Ferruzzi M.G., Böhm V., Courtney P.D., Schwartz S.J. (2002). Antioxidant and antimutagenic activity of dietary chlorophyll derivatives determined by radical scavenging and bacterial reverse mutagenesis assays. J. Food Sci..

[31] Koyande A.K., Chew K.W., Rambabu K., Tao Y., Chu D.-T., Show P.-L. (2019). Microalgae: A potential alternative to health supplementation for humans. Food Sci. Hum. Wellness.

[32] Roca M., Chen K., Pérez-Gálvez A., Carle R., Schweiggert R.M. (2016). Chlorophylls. Handbook on Natural Pigments in Food and Beverages Industrial Applications for Improving.

[33] Zhao W., Duan M., Zhang X., Tan T. (2018). A mild extraction and separation procedure of polysaccharide, lipid, chlorophyll and protein from *Chlorella* spp.. Renew. Energy.

[34] Queiroz Zepka L., Jacob-Lopes E., Roca M. (2019). Catabolism and bioactive properties of chlorophylls. Curr. Opin. Food Sci..

[35] Safi C., Zebib B., Merah O., Pontalier P.Y., Vaca-Garcia C. (2014). Morphology, composition, production, processing and applications of *Chlorella vulgaris*: A review. Renew. Sustain. Energy Rev..

[36] Liu J., Chen F., Posten C., Chen S.F. (2014). Biology and Industrial Applications of Chlorella: Advances and Prospects. Microalgae Biotechnology.

[37] Paniagua-Michel J. (2015). Microalgal Nutraceuticals. Handbook of Marine Microalgae.

[38] Pan-utai W., Kahapana W., Iamtham S. (2018). Extraction of C-phycocyanin from *Arthrospira* (*Spirulina*) and its thermal stability with citric acid. J. Appl. Phycol..

[39] Pagels F., Guedes A.C., Amaro H.M., Kijjoa A., Vasconcelos V. (2019). Phycobiliproteins from cyanobacteria: Chemistry and biotechnological applications. Biotechnol. Adv..

[40] Manirafasha E., Ndikubwimana T., Zeng X., Lu Y., Jing K. (2016). Phycobiliprotein: Potential microalgae derived pharmaceutical and biological reagent. Biochem. Eng. J..

[41] Dineshbabu G., Goswami G., Kumar R., Sinha A., Das D. (2019). Microalgae–nutritious, sustainable aqua- and animal feed source. J. Funct. Foods.

[42] Bhalamurugan G.L., Valerie O., Mark L. (2018). Valuable bioproducts obtained from microalgal biomass and their commercial applications: A review. Environ. Eng. Res..

[43] Soni R.A., Sudhakar K., Rana R.S. (2017). *Spirulina*–From growth to nutritional product: A review. Trends Food Sci. Technol..

[44] Juszkiewicz A., Basta P., Petriczko E., Machaliński B., Trzeciak J., Łuczkowska K., Skarpańska-Stejnborn A. (2018). An attempt to induce an immunomodulatory effect in rowers with spirulina extract. J. Int. Soc. Sports Nutr..

[45] Deng R., Chow T.-J. (2010). Hypolipidemic, Antioxidant and Antiinflammatory Activities of Microalgae *Spirulina*. Cardiovasc. Ther..

[46] Gad A.S., Khadrawy Y.A., El-Nekeety A.A., Mohamed S.R., Hassan N.S., Abdel-Wahhab M.A. (2011). Antioxidant activity and hepatoprotective effects of whey protein and *Spirulina* in rats. Nutrition.

[47] Wu Q., Liu L., Miron A., Klímová B., Wan D., Kuča K. (2016). The antioxidant, immunomodulatory, and anti-inflammatory activities of *Spirulina*: An overview. Arch. Toxicol..

[48] Wan D., Wu Q., Kuča K. (2016). *Spirulina*. Nutraceuticals.

[49] Pulz M.O., Gross W. (2004). Valuable products from biotechnology of microalgae. Appl. Microbiol. Biotechnol..

[50] De Morais M.G., Morais D., Greque E., da Silva Vaz B., Gonçalves C.F., Lisboa C., Vieira Costa J.A. (2016). Nanoencapsulation of the Bioactive Compounds of *Spirulina* with a Microalgal Biopolymer Coating. J. Nanosci. Nanotechnol..

[51] Food and Drug Aministration Listing of Color Additives Exempt Certification. https://www.accessdata.fda.gov/scripts/cdrh/cfdocs/cfcfr/CFRSearch.cfm?fr=73.530.

[52] İlter I., Akyıl S., Demirel Z., Koç M., Conk-Dalay M., Kaymak-Ertekin F. (2018). Optimization of phycocyanin extraction from *Spirulina platensis* using different techniques. J. Food Compos. Anal..

[53] Pan-utai W., Iamtham S. (2019). Extraction, purification and antioxidant activity of phycobiliprotein from *Arthrospira platensis*. Process Biochem..

[54] Nwoba E.G., Parlevliet D.A., Laird D.W., Alameh K., Moheimani N.R. (2019). Sustainable phycocyanin production from *Arthrospira platensis* using solar-control thin film coated photobioreactor. Biochem. Eng. J..

[55] Chen C.Y., Kao P.C., Tsai C.J., Lee D.J., Chang J.S. (2013). Engineering strategies for simultaneous enhancement of C-phycocyanin production and CO_2_ fixation with *Spirulina platensis*. Bioresour. Technol..

[56] Eriksen N.T. (2008). Production of phycocyanin—A pigment with applications in biology, biotechnology, foods and medicine. Appl. Microbiol. Biotechnol..

[57] Gong M., Bassi A. (2016). Carotenoids from microalgae: A review of recent developments. Biotechnol. Adv..

[58] Rammuni M.N., Ariyadasa T.U., Nimarshana P.H.V., Attalage R.A. (2019). Comparative assessment on the extraction of carotenoids from microalgal sources: Astaxanthin from *H. pluvialis* and β-carotene from *D. salina*. Food Chem..

[59] Soares A.T., da Costa D.C., Vieira A.A.H., Antoniosi Filho N.R. (2019). Analysis of major carotenoids and fatty acid composition of freshwater microalgae. Heliyon.

[60] Yoshida H., Yanai H., Ito K., Tomono Y., Koikeda T., Tsukahara H., Tada N. (2010). Administration of natural astaxanthin increases serum HDL-cholesterol and adiponectin in subjects with mild hyperlipidemia. Atherosclerosis.

[61] Tanaka T., Shnimizu M., Moriwaki H. (2012). Cancer chemoprevention by carotenoids. Molecules.

[62] Ye Z.W., Jiang J.G., Wu G.H. (2008). Biosynthesis and regulation of carotenoids in *Dunaliella*: Progresses and prospects. Biotechnol. Adv..

[63] Darvin M.E., Fluhr J.W., Meinke M.C., Zastrow L., Sterry W., Lademann J. (2011). Topical beta-carotene protects against infra-red-light-induced free radicals. Exp. Dermatol..

[64] Getrude N., Albert N., Gabriel G., Finbarrs-Bello E., Elizabeth C., Austin O. (2018). Beta-Carotene: Positive Effect on Oxidative Stress, Lipid Peroxidation, Insulin and Leptin Resistance Induced by Dietary Fat Consumption. J. Adv. Med. Med. Res..

[65] Tinoco N.A.B., Teixeira C.M.L.L., Rezende C.M. (2015). The Genus *Dunaliella*: Biotechnology and Applications. Rev. Virtual Química.

[66] Francavilla M., Trotta P., Luque R. (2010). Phytosterols from *Dunaliella tertiolecta* and *Dunaliella salina*: A potentially novel industrial application. Bioresour. Technol..

[67] Xu Y., Harvey P.J. (2019). Carotenoid production by *Dunaliella salina* under red light. Antioxidants.

[68] EU Commission (2012). European Comission Commission Regulation (EU) No 231/2012. Off. J. Eur. Union.

[69] Zuluaga M., Gueguen V., Letourneur D., Pavon-Djavid G. (2018). Astaxanthin-antioxidant impact on excessive Reactive Oxygen Species generation induced by ischemia and reperfusion injury. Chem. Biol. Interact..

[70] Li J., Zhu D., Niu J., Shen S., Wang G. (2011). An economic assessment of astaxanthin production by large scale cultivation of *Haematococcus pluvialis*. Biotechnol. Adv..

[71] Panis G., Carreon J.R. (2016). Commercial astaxanthin production derived by green alga *Haematococcus pluvialis*: A microalgae process model and a techno-economic assessment all through production line. Algal Res..

[72] Naguib Y.M.A. (2000). Antioxidant activities of astaxanthin and related carotenoids. J. Agric. Food Chem..

[73] Alghazwi M., Smid S., Musgrave I., Zhang W. (2019). In vitro studies of the neuroprotective activities of astaxanthin and fucoxanthin against amyloid beta (Aβ 1-42) toxicity and aggregation. Neurochem. Int..

[74] Hussein G., Sankawa U., Goto H., Matsumoto K., Watanabe H. (2006). Astaxanthin, a Carotenoid with Potential in Human Health and Nutrition. J. Nat. Prod..

[75] Guerin M., Huntley M.E., Olaizola M. (2003). *Haematococcus* astaxanthin: Applications for human health and nutrition. Trends Biotechnol..

[76] Shah M.M.R., Liang Y., Cheng J.J., Daroch M. (2016). Astaxanthin-producing green microalga *Haematococcus pluvialis*: From single cell to high value commercial products. Front. Plant Sci..

[77] Fang N., Wang C., Liu X., Zhao X., Liu Y., Liu X., Du Y., Zhang Z., Zhang H. (2019). De novo synthesis of astaxanthin: From organisms to genes. Trends Food Sci. Technol..

[78] Borowitzka M.A. (2018). Microalgal Metabolism and their Utilisation. Marine Macro- and Microalgae.

[79] Khoo K.S., Lee S.Y., Ooi C.W., Fu X., Miao X., Ling T.C., Show P.L. (2019). Recent advances in biorefinery of astaxanthin from *Haematococcus pluvialis*. Bioresour. Technol..

[80] European Comission Comission Implementing Regulation (EU) 2017/2470. https://eur-lex.europa.eu/legal-content/EN/TXT/?uri=uriserv:OJ.L_.2017.351.01.0072.01.ENG&toc=OJ:L:2017:351:TOC.

[81] Turck D., Castenmiller J., de Henauw S., Hirsch-Ernst K.I., Kearney J., Maciuk A., Mangelsdorf I., McArdle H.J., Naska A., Pelaez C. (2020). Safety of astaxanthin for its use as a novel food in food supplements. EFSA J..

[82] The Boeing Company (1964). Algae nutrient. Patent.

[83] Gudin C., Jungas C., Vaillant J. (1989). Method and Device for Producing Carotenoids and Particularly Astaxanthin by Culture of Microalga. Patent.

[84] Toshimitsu K., Shiyuusuke S. (1980). Preparation of Alcohollresistant Highhpurity Phycocyanin. Patent.

[85] Du W., Yu S., Yyu P., Zuo S. (2019). Sterilization Method of *Spirulina* Powder with High Phycocyanin Content. Patent.

[86] Cao B., Deng X., Gao K., Hu X., Li D., Yan R. (2019). Method for Increasing Content of *Chlorella* Chlorophyll. Patent.

[87] Xiao H., Zhang D. (2019). Method for Industrial Co-Production of Phycocyanin, *Spirulina* Polysaccharide and Protein Feed from Fresh Spirulina. Patent.

[88] Tang Y. (2019). Dyeing method of natural *Dunaliella salina* Beta-Carotene. Patent.

[89] Liu H., Wei C. (2019). *Haematococcus pluvialis* Tabletted Candies and Preparation Method thereof. Patent.

[90] Ishikawa E., Kasaba K., Shibata S., Shimura K., Shirota S. (2014). *Chlorella* with High Content of Chlorophyll and Carotenoid. Patent.

[91] Hong J.H., Kang I.J., Lee D., No H.K., Park H.M. (2014). Method for Producing *Chlorella* Powder with Increased Chlorophyll Content and anTioxidant Activity. Patent.

[92] Kitamura H., Katsumi Y. (2008). Green Color-based Beverage Composition. Patent.

[93] Koichi N. (2000). Chlorophyll-Rich and Salt-Resistant Chlorella. Patent.

[94] Wang Z., Zhang H., Zhu W. (2019). A Separation and Purification Method for High-Purity Phycocyanin. Patent.

[95] Li J., Ou Y., Yuan Z. (2014). Preparation Method of High-Purity Phycocyanin. Patent.

[96] Zhou Y. (2016). Extraction Process for Spirulina Phycocyanin and Use of *Spirulina* Phycocyanin. Patent.

[97] Zhang C., Wang Z., Liu Z. (1996). Blue Liquid *Spirulina* Beverage and Preparing Process Thereof. Patent.

[98] Jiang J., Liang M., Wan L. (2018). Method for Promoting Accumulation of Carotenoids and Beta-Carotene in *Dunaliella* by Using Beta-Ionone. Patent.

[99] Jin E.S., Kim M.J. (2017). *Dunaliella* Variant and Method for Producing Pigment by Using Same. Patent.

[100] Chang C.-M., Shen Y.-C., Chang C.M., Shen Y.C. (2013). Method for Preparing Purity Beta-Carotene Nano Powder from *Dunaliella salina*. Patent.

[101] Haigh W.G. (1994). High Purity Beta-Carotene. Patent.

[102] Hong Y., Wang P. (2019). Method for Rapidly Extracting Astaxanthin from *Haematococcus pluvialis*. Patent.

[103] Gao M., Qian W., Wang L., Zhu X. (2019). Method for Inducing Efficient Accumulation of Astaxanthin in *Haematococcus pluvialis* by Adding Nanometer Material. Patent.

[104] Duan M., Fang T., Ge F., Ma J., Shi Q. (2018). Preparation Method of Antioxidant Astaxanthin Soft Capsules. Patent.

[105] Choi Y.E., Lee C.S. (2018). Method to Enhance the Astaxanthin Biosynthesis in Microalga *Haematococcus pluvialis*. Patent.

[106] QYResearch (2019). Global Algae Market Report, History and Forecast (2014–2025), Breakdown Data by Manafacturers, Key Regions, Types and Application. Algae Systems, Sapphire Energy, Solazyme. QYResearch.

[107] Baehr L. The Next Big Superfood Could Be Green and Slimy. https://www.businessinsider.com/algae-is-the-superfood-of-the-future-2014-6.

[108] Bhattacharya M., Goswami S. (2020). Microalgae—A green multi-product biorefinery for future industrial prospects. Biocatal. Agric. Biotechnol..

[109] Allied Market Research (2019). Spirulina Market by Type (Arthrospira Platensis and Arthrospira Maxima), Application (Nutraceuticals, Cosmetics, Food & Beverages, Animal Feed, and Others), and Drug Formulation (Powder, Tablet & Capsule, Liquid, and Granule & Gelling Agent): Global Oppor. Allied Market Research.

[110] Future Market Insights (2018). Phycocyanin Market Growing at a CAGR of 7.6% During the Forecast Period, 2018–2028. Future Market Insights.

[111] Mohammadi-Gouraji E., Soleimanian-Zad S., Ghiaci M. (2019). Phycocyanin-enriched yogurt and its antibacterial and physicochemical properties during 21 days of storage. LWT.

[112] Chentir I., Kchaou H., Hamdi M., Jridi M., Li S., Doumandji A., Nasri M. (2019). Biofunctional gelatin-based films incorporated with food grade phycocyanin extracted from the Saharian cyanobacterium *Arthrospira* sp.. Food Hydrocoll..

[113] Market Research Future (2019). Global Chlorella Market Research Report: By Type (*Chlorella vulgaris*, *Chorella pyrenoidosa*, *Chlorella ellipsoidea* and others), Application (Functional Food & Beverages, Nutraceuticals & Pharmaceuticals, Personal Care, others), and Region (North America). Market Research Future.

[114] Gouveia L., Batista A.P., Miranda A., Empis J., Raymundo A. (2007). *Chlorella vulgaris* biomass used as colouring source in traditional butter cookies. Innov. Food Sci. Emerg. Technol..

[115] Value Market Research (2018). Global Chlorophyll Extract Market Report 2018–2025. Value Market Research.

[116] Ohi Y., Namiki T., Katatae M., Tsukahara H., Kitamura A. (2009). Effects of the Addition of the Natural Carotenoid Astaxanthin from Microalgae *Haematococcus pluvialis* on the Physical Properties of Bread. Nippon Shokuhin Kagaku Kogaku Kaishi.

[117] Borowitzka M.A. (2013). Dunaliella: Biology, Production, and Markets. Handbook of Microalgal Culture: Applied Phycology and Biotechnology.

[118] Global Market Insights (2019). Astaxanthin Market Size by Source (Synthetic, Natural), By Application (Dietary Supplement, Personal Care, Pharmaceuticals, Food & Beverages, Animal Feed {Aquaculture, Livestock, Pets}) Industry Outlook Report, Regional Analysis, Application Potential, Pr. Global Market Insights.

[119] Market Watch (2020). Beta Carotene Market 2018–2026|Industry Analysis, Size, Share, Growth, Trends and Forecast. Market Watch.

[120] Mulders K.J.M., Lamers P.P., Martens D.E., Wijffels R.H. (2014). Phototrophic pigment production with microalgae: Biological constraints and opportunities. J. Phycol..

[121] Aria M., Cuccurullo C. (2017). bibliometrix: An R-tool for comprehensive science mapping analysis. J. Informetr..

[122] Van Eck N.J., Waltman L. (2010). Software survey: VOSviewer, a computer program for bibliometric mapping. Scientometrics.

